# Supplementary material for: Loss of Fam60a, a Sin3a subunit, results in embryonic lethality and is associated with aberrant methylation at a subset of gene promoters
Source: eLife. 2018 Aug 2;7:e36435. doi: 10.7554/eLife.36435 (PMC6072441; doi:10.7554/eLife.36435)
Supplement: Supplementary file 1. [file elife-36435-supp1.docx]

Figure 3-supplement table 1

Distribution of Fam60a genotypes for mouse embryos obtained by heterozygote intercrosses at various stages of development.

| Stage | *Fam60a^+/+^* | *Fam60a^+/-^* | *Fam60a^-/-^* | Total |
| --- | --- | --- | --- | --- |
| E9.5 | 80 (23.3%) | 174 (50.6%) | 90 (26.2%) | 344 |
| E10.5 | 39 (27.5%) | 63 (44.4%) | 40 (28.2%) | 142 |
| E11.5 | 23 (23.0%) | 59 (59.0%) | 18 (18.0%) | 100 |
| E12.5 | 16 (22.9%) | 42 (60.0%) | 12 (17.1%) | 70 |
| E13.5 | 14 (21.9%) | 38 (59.4%) | 12 (18.8%) | 64 |
| E18.5 | 18 (28.6%) | 41 (65.1%) | 4 (6.3%) | 63 |
