# Supplementary material for: Loss of Fam60a, a Sin3a subunit, results in embryonic lethality and is associated with aberrant methylation at a subset of gene promoters
Source: eLife. 2018 Aug 2;7:e36435. doi: 10.7554/eLife.36435 (PMC6072441; doi:10.7554/eLife.36435)
Supplement: Supplementary file 2. [file elife-36435-supp2.docx]

| Library | Methylated CpGs | | Unmethylated CpGs | |
| --- | --- | --- | --- | --- |
| *Fam60a^+/+^* embryo 1 | 55,301,002 | 45.8% | 65,324,110 | 54.2% |
| *Fam60a^+/+^* embryo 2 | 43,690,864 | 46.0% | 51,193,421 | 54.0% |
| *Fam60a^+/+^* embryo 3 | 56,201,851 | 44.2% | 70,981,705 | 55.8% |
| *Fam60a^-/-^* embryo 1 | 57,123,330 | 45.9% | 67,350,896 | 54.1% |
| *Fam60a^-/-^* embryo 2 | 51,987,177 | 45.2% | 63,117,178 | 54.8% |
| *Fam60a^-/-^* embryo 3 | 51,817,415 | 44.1% | 65,716,556 | 55.9% |

Figure 8-supplement table 1

Methylation level of CpG sites in the captured DNA in three embryos of each genotype.
